# Stress and Depressive Symptoms in Cancer Survivors and Their Family Members: Korea Community Health Survey, 2012

**DOI:** 10.3390/ijerph14090999

**Published:** 2017-09-01

**Authors:** Mi Ah Han

**Affiliations:** Department of Preventive Medicine, College of Medicine, Chosun University, Gwangju 61452, Korea; mahan@chosun.ac.kr; Tel.: +82-62-230-6481

**Keywords:** chronic disease, depression, family, neoplasms, stress, survivors

## Abstract

This study examined the prevalence of perceived stress and depressive symptoms in cancer survivors and their family members compared with subjects without cancer and without family members with cancer. The subjects of this cross-sectional study were adults ≥19 years old who participated in the 2012 Korea Community Health Survey. Stress and depressive symptoms in cancer survivors and their family members were assessed and compared to symptoms in control groups by chi-square tests and multiple logistic regression analyses. Of the 6783 cancer survivors, 26.9% and 8.7% reported having stress and depressive symptoms, respectively, and 27.7% and 5.9% of family members of cancer survivors reported having stress and depressive symptoms, respectively. Cancer survivors showed higher adjusted odds ratio (aOR) for stress (aOR = 1.26, 95% confidence interval (CI) = 1.16–1.37) and depressive symptoms (aOR = 1.82, 95% CI = 1.57–2.11) than subjects without cancer history. Family members of cancer survivors showed a higher OR for stress and depressive symptoms than subjects without a family member who survived cancer. Cancer survivors and family members of cancer survivors had more stress and depressive symptoms than controls. Careful management for cancer patients and their family members should include screening for stress and depression to improve mental health associated with cancer survivorship.

## 1. Introduction

About 14.1 million new cancer cases and 8.2 million cancer-associated deaths occurred worldwide in 2012, and the occurrence of cancer is steadily increasing [1]. In Korea, the number of cancer patients is continuously increasing, and the probability of survival is simultaneously increasing [2]. Despite significant advances, cancer and cancer treatments can result in significant physical and emotional morbidity, and several epidemiological studies indicate that the risk of poor mental health is elevated in cancer patients compared with the general population [3,4,5,6,7]. Cancer patients experience several stressors and suffer from depression [8,9]. Depression is the most important risk factor for suicide [10,11,12]. Subjects with a history of cancer had a higher odds ratio (OR) for attempted suicide in both genders using the Third US National Health and Nutrition Examination Survey [4]. Thus, understanding the prevalence and correlation of stress and depression with cancer is crucial for the management and control of mental health.

Family members of cancer patients also are at risk for psychological problems and are more susceptible to depression [13]. Family caregivers with anxiety or depression also have a high risk of suicide [14].

This study aimed to describe stress and depressive symptoms among cancer survivors and their family members in Korea. We compared stress and depressive symptoms of cancer survivors and their family members with two control populations (non-cancer controls, non-cancer non-family member controls) using the 2012 Korea Community Health Survey (KCHS, 2012).

## 2. Materials and Methods

### 2.1. Data Source and Study Subjects

The 2012 KCHS is a cross-sectional survey conducted by the Korea Centers for Disease Control and Prevention that collected information related to health status, health care utilization, and health determinants for Korean individuals living in private occupied dwellings in 254 regional sites covering all provinces and territories. Using a multistage stratified cluster-sampling procedure, the 2012 KCHS surveyed 228,921 household residents 19 years of age and over. Detailed information about the KCHS has been documented elsewhere [15].

Participants were classified as cancer survivors if they reported having ever been told by a doctor that they had any kind of cancer. Family members of cancer survivors were defined as subjects who lived with cancer survivors during the survey time. Non-cancer and non-family member controls were selected among subjects without cancer and without family members with cancer with 1:1 individual matching on sex and age using matched simple random sampling methods. Subjects who were living alone were excluded. Therefore, the final study subjects included 6783 cancer survivors, 6783 non-cancer controls, 8585 family members of cancer survivors, and 8585 non-cancer, non-family controls.

### 2.2. Stress and Depressive Symptoms

Perceived stress, consultation experience due to stress, depressive symptoms, and consultation experience due to depressive symptoms were collected. Stress was defined as the responses “very much” or “much” to the following question: “How much do you feel stress in your usual life?” Depressive symptoms was defined as a “yes” response to the following question: “During the past 12 months, did you feel so much sadness/hopelessness for two or more weeks that you stopped doing some usual activities?” Consultation experience was defined as professional consultation (medical institute, professional consultation office, community health center, etc.) due to stress or depressive symptoms.

### 2.3. Covariates

Covariates included sex, age, marital status (with spouse, without spouse), education (uneducated, elementary school, middle school, high school, at least some college), smoking status (never, ex, current), drinking frequency (none, ≤1/month, ≥2/month), number of chronic disease (none, 1, 2 or more), unmet needs for health care (no, yes), and utilization of community health service (no, yes). The chronic diseases included were hypertension, diabetes, dyslipidemia, stroke, myocardial infarction, osteoarthritis, osteoporosis, and tuberculosis. Having unmet needs for health care was defined as a “yes” response to the following question: “During the past 12 months, was there any time when you did not get the medical care you needed?” When subjects reported “yes” response, the reasons for unmet needs were asked: (1) cost; (2) reservation problems; (3) transportation problems; (4) not available at the required time; (5) waiting time too long; (6) mild symptoms; (7) other.

### 2.4. Statistical Analysis

Descriptive statistics and chi-square tests were used to examine differences in demographic characteristics between cancer survivors, their family members, and the controls. Chi-square tests and logistic regression models were used to investigate whether stress and depressive symptoms differed between cancer survivors, family members, and controls. Finally, the associated factors for stress and depressive symptoms in cancer survivors and family members were investigated using multiple logistic regression analysis. All data analyses were performed using SAS 9.2 software (SAS Institute, Cary, NC, USA). Differences were considered statistically significant at *p* < 0.05.

## 3. Results

Cancer survivors and non-cancer controls showed significant differences in general characteristics. The proportions of current smokers and individuals drinking ≥2/month were lower in cancer survivors than in non-cancer controls. There were significant differences between family members and controls in the number of chronic illnesses (Table 1).

Among 6783 cancer survivors, 26.9% and 8.7% reported stress and depressive symptoms, respectively. Cancer survivors were more likely to have stress (adjusted odds ratio (aOR) = 1.26, 95% confidence interval (CI) = 1.16–1.37), consultation experience due to stress (aOR = 1.57, 95% CI = 1.21–2.03), depressive symptoms (aOR = 1.82, 95% CI = 1.57–2.11), and consultation experience due to depressive symptoms (aOR = 1.86, 95% CI = 1.32–2.64). In addition, 27.7% and 5.9% of cancer survivor family members reported stress and depressive symptoms, respectively. The risks for stress (aOR = 1.08, 95% CI = 1.01–1.16) and depressive symptoms (aOR = 1.31, 95% CI = 1.14–1.51) were significantly higher in family members of cancer survivors than controls without cancer and without family members with cancer (Table 2).

Stress was associated with gender, age, education, smoking, number of chronic disease, unmet needs for health care, and utilization of community health service in cancer survivors. Depressive symptoms were associated with gender, marital status, smoking, alcohol drinking, number of chronic diseases, and unmet needs for health care in cancer survivors. Similar associations were shown in family members of cancer survivors (Table 3).

## 4. Discussion

In this study, we described the stress and depressive symptoms of cancer survivors and their family members, and compared these symptoms with controls. Among 6783 cancer survivors, 26.9% of survivors were stressed, and 8.7% of survivors had depressive symptoms. Cancer survivors were more likely to have stress and depressive symptoms compared with controls. Similar results were found in family members of survivors.

Cancer survivors have a high incidence of stress and depressive symptoms compared with controls, which are risk factors for suicide in cancer survivors [16]. Unfortunately, depression and psychosocial stresses associated with cancer often go unrecognized or underestimated, leaving many patients untreated. Previous studies reported that among 144 patients with gynecologic or breast cancer who were diagnosed with major depression, only 12% reported receiving antidepressants, and only 5% reported seeing a counselor or participating in a cancer support group [17]. Compared with healthy people and those with benign diseases, cancer patients report significantly depressed mood that persists up to four months after diagnosis and during anticipation of treatment [18]. These results suggest that careful monitoring of psychotic aspects of cancer patients is as important as their cancer management.

Family members of cancer survivors were more likely to have stress and depressive symptoms than controls. In this study, the prevalence of depressive symptoms among family members of cancer survivors was 5.9%, which was lower than that among caregivers of stroke survivors (40.2%) [19] and Alzheimer disease (34.0%) [20]. There are several possible explanations for these results. First, stroke survivors and patients with dementia are more likely to exhibit cognitive impairments and behavioral problems. As a result, their caregivers are more likely to develop depression. Second, the mortality rate of cancer patients may be higher than that of stroke survivors and dementia. The duration and burden of caregiving for cancer patients may be shorter and less than those of looking after stroke survivors and patients with dementia. Diagnosis of cancer is a stressful event for family members as well as cancer patients [21]. In previous studies, the prevalence of stress and depression among family member of cancer patients was high [22], and the perceived burden is the best predictor of depression [23]. Recognition of cancer diagnosis, caring for patients, medical expenses, and the threat of death can lead to psychological problems in family members [24]. These psychological effects of cancer can be a threat to the physical health of family members, thus efforts to reduce the psychological effects of cancer should focus not only on the patient but also on the family members. Smartphone applications can be applied to improve after-care of cancer survivors [25] and provides psychoeducation to caregivers [26] based on previous applications on stroke survivors and dementia.

Women cancer survivors and family members were more likely to report stress and depressive symptoms. This was consistent with previous study in Korea which showed that women cancer survivors were more likely to experience depression than men [27]. Higher psychological problems were reported among women cancer patients. They had a tendency to be more depressed and anxious than men with cancer [28] and were more likely to express their feelings and report more symptoms than men [29].

Although the unmet needs for health care in cancer survivors and family member were not higher than those of controls, they were significantly associated with stress and depressive symptoms in this study. Previous studies revealed that unmet needs were associated with low quality of life in cancer patients [30] and were positively associated with depression among caregivers of cancer patients [31]. These results suggested that monitoring and offering support for adequate health care needs of cancer survivors and family member are important for reducing stress and depressive symptoms.

There are several limitations to using KCHS data to examine stress and depressive symptoms in cancer survivors and their family members. First, because of the cross-sectional nature of this data, we were unable to examine stress and depressive symptoms before cancer diagnosis. Stress and depression have been considered as risk factors for cancer development [32]. Second, the KCHS does not include information about cancer type or current cancer status. Thus, we were unable to determine the proportion of cancer survivors who were actively dealing with treatment or recurrent/advanced disease versus those living disease- and/or symptom-free. Third, cancer survivors who lived in nursing homes, long-term care facilities, or hospitals were not included in the survey, so the results of this study might not accurately reflect the status of all cancer patients in Korea. Fourth, reliability and validity for the single-item surveys on stress and depressive symptoms were not investigated. However, the single-item measure of stress or depression was suggested to be a valid measure for group level data or screening for psychological status [33,34]. Finally, in this cross-sectional study, there was potential for selection bias because of selective survival, as individuals who had been diagnosed with cancer and subsequently died before the survey would not have had the opportunity to participate in the study.

## 5. Conclusions

This study provides population-based measures of stress and depressive symptoms among cancer survivors and their family members in Korea. Cancer survivors were more likely to have stress and depressive symptoms than individuals without a cancer history. Also, family members of cancer survivors reported more stress and depressive symptoms compared with controls. Screening or strategies to control or reduce psychological issues associated with cancer should be considered both for cancer survivors and their family members.

## Figures and Tables

**Table 1:** Characteristics of cancer survivors, family members, and controls.

Characteristics	Cancer Survivors (*n* = 6783)	Non-Cancer Controls (*n* = 6783)	*p*	Family Members of Cancer Survivors (*n* = 8585)	Non-Cancer Non-Family Controls (*n* = 8585)	*p*
Gender			1.000			1.000
Male	3157 (46.5)	3157 (46.5)		4148 (48.3)	4148 (48.3)	
Female	3626 (53.5)	3626 (53.5)		4437 (51.7)	4437 (51.7)	
Age (years)			1.000			1.000
18–39	698 (10.3)	698 (10.3)		2864 (33.4)	2864 (33.4)	
40–64	2952 (43.5)	2952 (43.5)		3139 (36.6)	3139 (36.6)	
≥65	3133 (46.2)	3133 (46.2)		2582 (30.1)	2582 (30.1)	
Marital status			0.258			0.145
Without spouse	894 (13.2)	939 (13.8)		2371 (27.6)	2286 (26.6)	
With spouse	5889 (86.8)	5844 (86.2)		6214 (72.4)	6299 (73.4)	
Education			0.259			0.654
Uneducated	1002 (14.8)	1072 (15.8)		1080 (12.6)	1144 (13.3)	
Elementary school	1770 (26.1)	1818 (26.8)		1626 (18.9)	1594 (18.6)	
Middle school	1180 (17.4)	1125 (16.6)		1000 (11.7)	1006 (11.7)	
High school	1773 (26.1)	1752 (25.8)		2715 (31.6)	2711 (31.6)	
≥College	1058 (15.6)	1016 (15.0)		2164 (25.2)	2130 (24.8)	
Smoking			<0.001			0.945
Never	4137 (61.0)	4196 (61.9)		5324 (62.0)	5338 (62.2)	
Ex	2041 (30.1)	1546 (22.8)		1448 (16.9)	1452 (16.9)	
Current	605 (8.9)	1041 (15.4)		1813 (21.1)	1795 (20.9)	
Drinking frequency			<0.001			0.202
None	3892 (57.4)	2891 (42.6)		3068 (35.7)	3119 (36.3)	
≤1/month	1264 (18.6)	1524 (22.5)		2105 (24.5)	2005 (23.4)	
≥2/month	1627 (24.0)	2368 (34.9)		3412 (39.7)	3461 (40.3)	
Number of diseases			0.007			0.044
None	2853 (42.1)	3008 (44.4)		4714 (54.9)	4818 (56.1)	
1	1928 (28.4)	1922 (28.3)		1863 (21.7)	1896 (22.1)	
≥2	2002 (29.5)	1853 (27.3)		2008 (23.4)	1871 (21.8)	
Unmet needs for health care			0.007			0.150
No	6120 (90.2)	6023 (88.8)		7588 (88.4)	7525 (87.7)	
Yes	663 (9.8)	760 (11.2)		997 (11.6)	1058 (12.3)	
Reasons for unmet needs ^a^						
Cost	223 (33.6)	208 (27.4)		232 (23.3)	238 (22.5)	
Reservation problems	25 (3.8)	15 (2.0)		24 (2.4)	22 (2.1)	
Transportaion problems	75 (11.3)	81 (10.7)		72 (7.2)	92 (8.7)	
Not available at time required	162 (24.4)	229 (30.1)		385 (38.7)	364 (34.4)	
Waiting time too long	30 (4.5)	29 (3.8)		39 (3.9)	39 (3.7)	
Mild symptoms	72 (10.9)	112 (14.7)		150 (15.1)	170 (16.1)	
Other	76 (11.5)	86 (11.3)		94 (9.4)	133 (12.6)	
Community health service utilization			0.882			0.276
No	6302 (55.8)	3563 (55.7)		5272 (64.5)	5304 (65.3)	
Yes	2850 (44.2)	2834 (44.3)		2904 (35.5)	2819 (34.7)	

Data are expressed as number (%). ^a^ Limited to subjects who have the unmet health care needs.

**Table 2:** Stress and depressive symptoms in cancer survivors, family members, and controls.

Characteristics	Cancer Survivors	Controls	*p*	Family Members of Cancer Survivors	Non-Cancer Non-Family Controls	*p*
Stress			<0.001			0.015
No	4957 (73.1)	5245 (77.3)		6206 (72.3)	6348 (73.9)	
Yes	1826 (26.9)	1538 (22.7)		2379 (27.7)	2237 (26.1)	
cOR (95% CI)	1.26 (1.16–1.36)	1.00		1.09 (1.02–1.17)	1.00	
aOR (95% CI) ^a^	1.26 (1.16–1.37)	1.00		1.08 (1.01–1.16)	1.00	
Consultation due to stress			<0.001			0.603
No	6625 (97.7)	6682 (98.5)		8428 (98.2)	8437 (98.3)	
Yes	158 (2.3)	101 (1.5)		157 (1.8)	148 (1.7)	
cOR (95% CI)	1.58 (1.23–2.04)	1.00		1.06 (0.85–1.33)	1.00	
aOR (95% CI) ^a^	1.57 (1.21–2.03)	1.00		1.05 (0.84–1.32)	1.00	
Depressive symptoms			<0.001			<0.001
No	6194 (91.3)	6471 (95.4)		8082 (94.1)	8203 (95.6)	
Yes	589 (8.7)	312 (4.6)		503 (5.9)	382 (4.5)	
cOR (95% CI)	1.98 (1.72–2.28)	1.00		1.34 (1.17–1.54)	1.00	
aOR (95% CI) ^b^	1.82 (1.57–2.11)	1.00		1.31 (1.14–1.51)	1.00	
Consultation due to depressive symptoms			<0.001			0.378
No	6676 (98.4)	6732 (99.3)		8501 (99.0)	8512 (99.2)	
Yes	107 (1.6)	51 (0.8)		84 (1.0)	73 (0.9)	
cOR (95% CI)	2.12 (1.52–2.97)	1.00		1.15 (0.84–1.58)	1.00	
aOR (95% CI) ^b^	1.86 (1.32–2.64)	1.00		1.10 (0.80–1.52)	1.00	

Data are expressed as numbers (%). aOR, adjusted odds ratio; cOR, crude odds ratio; CI, confidence interval. ^a^ Adjusted for marital status, education, smoking, alcohol drinking, and number of chronic diseases; ^b^ Adjusted for marital status, education, smoking, alcohol drinking, number of chronic diseases, and perceived stress.

**Table 3:** Stress and depressive symptoms by characteristics in cancer survivors and family members.

Characteristics	Cancer Survivors				Family Members of Cancer Survivors			
	Stress		Depressive Symptoms		Stress		Depressive Symptoms	
	%	aOR (95% CI)	%	aOR (95% CI)	%	aOR (95% CI)	%	aOR (95% CI)
Total	26.9		8.7		27.7		5.9	
Gender								
Male	24.4	1.00	7.2	1.00	24.3	1.00	3.8	1.00
Female	29.1	1.47 (1.20–1.79)	10.0	1.42 (1.03–1.96)	30.9	1.77 (1.50–2.08)	7.8	2.56 (1.81–3.61)
Age (years)								
18–39	29.7	1.65 (1.29–2.11)	8.5	1.22 (0.82–1.82)	31.2	2.43 (1.96–3.00)	5.1	1.33 (0.88–1.99)
40–64	27.4	1.28 (1.11–1.48)	8.4	1.05 (0.84–1.32)	27.8	1.53 (1.31–1.79)	6.3	1.27 (0.96–1.68)
≥65	25.9	1.00	9.0	1.00	23.9	1.00	6.2	1.00
Marital status								
Without spouse	29.1	0.94 (0.79–1.12)	13.7	1.53 (1.20–1.95)	27.6	0.75 (0.66–0.86)	5.7	1.11 (0.85–1.44)
With spouse	26.6	1.00	7.9	1.00	27.8	1.00	5.9	1.00
Education								
Uneducated	32.2	1.31 (1.04–1.65)	9.9	0.87 (0.59–1.28)	29.8	1.20 (0.96–1.50)	7.9	1.06 (0.70–1.61)
Elementary school	28.4	1.15 (0.95–1.40)	9.8	1.17 (0.85–1.61)	28.7	1.07 (0.89–1.29)	6.8	0.90 (0.63–1.30)
Middle school	25.2	0.92 (0.74–1.13)	9.5	1.17 (0.84–1.64)	22.4	0.72 (0.59–0.89)	6.4	1.03 (0.71–1.50)
High school	23.6	0.81 (0.67–0.97)	7.2	0.84 (0.62–1.14)	26.5	0.84 (0.74–0.96)	5.1	0.86 (0.65–1.14)
≥College	26.8	1.00	7.3	1.00	29.9	1.00	4.9	1.00
Smoking								
Never	26.8	1.00	9.1	1.00	28.3	1.00	6.7	1.00
Ex	24.7	1.44 (1.17–1.76)	7.4	1.28 (0.92–1.78)	22.2	1.25 (1.02–1.52)	4.4	1.55 (1.04–2.31)
Current	34.9	2.13 (1.67–2.72)	10.4	1.75 (1.19–2.57)	30.5	1.81 (1.52–2.16)	4.7	1.63 (1.12–2.37)
Drinking frequency								
None	28.2	1.10 (0.95–1.27)	10.3	1.68 (1.30–2.17)	27.2	0.94 (0.82–1.08)	7.1	1.12 (0.86–1.46)
≤1/month	24.0	0.85 (0.71–1.03)	7.0	1.11 (0.80–1.53)	28.7	0.98 (0.86–1.13)	5.5	0.96 (0.73–1.26)
≥2/month	26.2	1.00	6.0	1.00	27.6	1.00	4.9	1.00
Number of diseases								
None	24.0	1.00	7.1	1.00	26.9	1.00	4.5	1.00
1	25.9	1.16 (1.01–1.34)	8.1	1.10 (0.87–1.40)	25.6	1.18 (1.02–1.36)	6.2	1.48 (1.12–1.96)
≥2	32.1	1.48 (1.28–1.72)	11.5	1.51 (1.19–1.91)	31.7	1.67 (1.43–1.94)	8.7	2.13 (1.60–2.82)
Unmet needs for health care								
No	24.5	1.00	7.4	1.00	25.1	1.00	4.9	1.00
Yes	49.5	3.00 (2.38–3.79)	21.0	3.21 (2.38–4.34)	47.6	2.44 (2.04–2.92)	13.0	3.03 (2.30–3.98)
Community health service utilization								
No	27.8	1.14 (1.01–1.29)	8.6	1.05 (0.86–1.29)	28.5	1.09 (0.97–1.23)	5.7	1.04 (0.83–1.31)
Yes	23.5	1.00	7.4	1.00	23.5	1.00	5.3	1.00

aOR, adjusted odds ratio; CI, confidence interval.
